# Association between promoter DNA methylation and gene expression in the pathogenesis of ischemic stroke

**DOI:** 10.18632/aging.102278

**Published:** 2019-09-17

**Authors:** Guo-Xiong Deng, Ning Xu, Qi Huang, Jin-Yue Tan, Zhao Zhang, Xian-Feng Li, Jin-Ru Wei

**Affiliations:** 1 Department of Cardiology, The First People’s Hospital of Nanning City, Nanning, Guangxi 530021, China; 2 Department of Neurology, The First People’s Hospital of Nanning City, Nanning, Guangxi 530021, China

**Keywords:** ischemic stroke, DNA methylation-mRNA expression-IS interaction network, function enrichment, correlation analyses

## Abstract

To assess DNA methylation sites as well as gene expression related to ischemic stroke (IS) and comprehensively reveal their correlation and possible pathological mechanisms, we implemented (1) genome-wide DNA methylation profiling from the GEO repository related to IS with and without symptoms; (2) identification of differentially methylation positions (DMPs) and genes (DMGs), functional enrichment analysis along with DMG regulatory network construction; (3) validation tests of 2 differential methylation positions of interest as well as analogous gene expression in other datasets and in IS patients and controls; and (4) correlation analysis of DNA methylation and mRNA expression data. In total, 870 DMPs were physically located within 693 DMGs. After disease ontology (DO), Kyoto Encyclopedia of Genes and Genomes (KEGG) pathway, gene ontology (GO), protein-protein interaction (PPI) network construction as well as module analysis, HLA-DRB1 and HLA-DQB1 were identified. Their expression was validated in 4 other datasets but was significant in only 1, and the expression was lower in the IS group (*P* < 0.05). After validation in IS patients and controls, we found that these two genes showed more hypermethylation and lower expression levels in the IS group (*P* < 0.001). The methylation of genes was negatively associated with their expression (*P* < 0.05). The current study recognized a connection among DNA methylation and gene expression and emphasized the prominence of HLA-DRB1 and HLA-DQB1 in IS pathogenesis.

## INTRODUCTION

Stroke, which kills more than 2 million people every year in China, is the second leading cause of death [1]. According to the different causes, stroke can be divided into several subtypes, including transient ischemic attack (TIA), cerebral infarction and hemorrhage [2]. Most strokes are caused by ischemic stroke (IS) and cerebral embolism, but not every patient has symptoms. Some patients have transient symptoms due to TIA [3]. Previous studies have shown that there are many major risk factors for cerebral ischemic infarction, including hypertension, diabetes, early family history, and other atherosclerosis-related diseases, such as hyperlipidemia [4]. According to the latest epidemiological studies, approximately 10 to 15% of strokes occur in young people aged 18 to 49 [5]. Therefore, a detailed understanding of the pathogenesis of ischemic stroke can provide a detailed theoretical basis for treatment.

In recent years, with the continuous improvement of research technology, there is a new understanding of the relationship between epigenetics and disease, and DNA methylation is a very important field in epigenetics research [6]. DNA methylation usually occurs on CpG islands, mostly in the proximal promoter region of the human genome [7]. DNA methylation alters an individual’s biological function by regulating gene expression or genome sequence stability [8]. It can keep transcription factors out of two gene promoters, inhibit transcription factor binding and change chromatin structures. Gene promoters might be available to vital cis-acting regulatory elements that initiate and control gene expression [9]. Methylation usually occurs rapidly and can usually be observed before the onset of disease. This important finding indicates that DNA methylation can also be used as an indicator of early screening for early or potential diseases [10].

At present, some studies have confirmed that abnormal methylation of gene promoters is associated with IS [11]. To classify new IS-related DNA methylation sites, we incorporated several microarray datasets from the Gene Expression Omnibus (GEO) repository and carried out analysis as well as validation to analyze the probable DNA methylation–mRNA expression–IS regulatory impact.

## RESULTS

### Data preprocessing and identified DMPs

**Figure 1:**
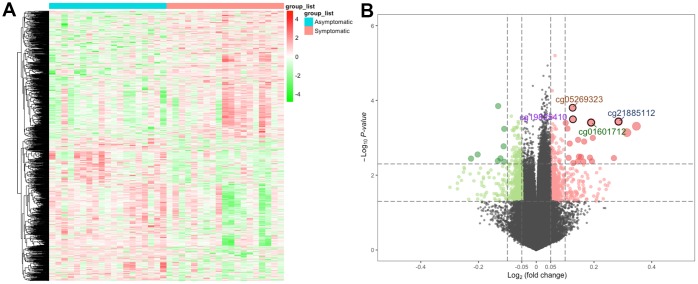
**The heatmap and volcano plot for DMPs.** (**A**) For the heatmap, the red strip represents symptomatic samples and the green strip represents asymptomatic samples. (**B**) For the volcano plot, the two vertical lines are the 0.05-fold change boundaries, and the horizontal line is the statistical significance boundary (*P* < 0.05). Items with statistical significance as well as hypermethylation are presented as red dots, and hypomethylation is presented as green dots in the volcano plot.

First, we determined DNA methylation levels at 485578 methylation sites in carotid plaques in GSE66500. After quality control and screening, 20019 methylation positions were subjected to differential analysis. In total, 1290 DMPs (|Δβ| > 0.05 and detection *P* < 0.05), including 608 hypermethylated and 682 hypomethylated DMPs, were recognized. As per the annotation, 870 DMPs were actually found within 693 unique genes (DMGs). The heatmap and volcano plot of the DMPs are presented in Figure 1. The details of these 870 DMPs can be found in Supplementary Table 1.

**Figure 2:**
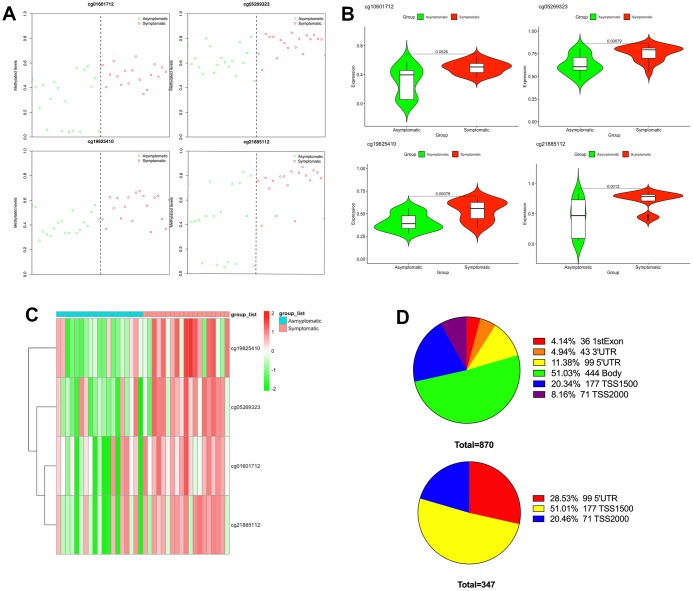
**Differential methylation between symptomatic and asymptomatic samples.** (**A**) Methylation levels of the 4 CpGs in asymptomatic (green circle) as well as symptomatic (red circle) samples from the GEO; (**B**) The differences in methylation levels of the 4 CpGs in asymptomatic and symptomatic patients; (**C**) Heat map indicating methylation of the 4 CpGs in asymptomatic and symptomatic patients; (**D**) Promoter region distribution of differentially methylated promoter CpG sites.

Subsequently, to recognize a set of CpGs that may differentiate symptomatic from asymptomatic patients, depending on the differentially methylated CpGs, we implemented shrunken centroid classifier analysis and found 4 hypermethylated CpGs in symptomatic patients (cg01601712, cg05269323, cg19825410 and cg21885112) that best discriminated between patients with and without symptoms (Figure 2A). These four differentially methylated loci correspond to three genes in the genome. The details can be found in Table 1. Then, we analyzed these four hypermethylated CpG sites and found that there were significant differences between them (*P* < 0.01–0.001) (Figure 2B). The methylation of these four differentially methylated CpG sites in the dataset is shown in Figure 2C. The proportion of differentially methylated CpG sites in the whole genome and the proportion of CpG island distribution of promoter differentially methylated CpG sites are displayed in Figure 2D.

**Table 1:** The details for DMPs.

**SYMBOL**	**CpG site**	**MAPINFO**	**CHR**	**Δβ**	***P* values**
HLA-DQB1	cg01601712	32635948	6	1.90E-01	3.91E-04
REPIN1	cg05269323	150067712	7	1.26E-01	1.57E-04
-	cg19825410	106092151	14	1.27E-01	3.21E-04
HLA-DRB1	cg21885112	32557970	6	2.85E-01	3.68E-04

*CHR*: chromosome; *Δ*β: difference of methylation between symptomatic patients and asymptomatic controls; *DMP*: differential methylation position; *MAPINFO*: position in Build 37.

**Figure 3:**
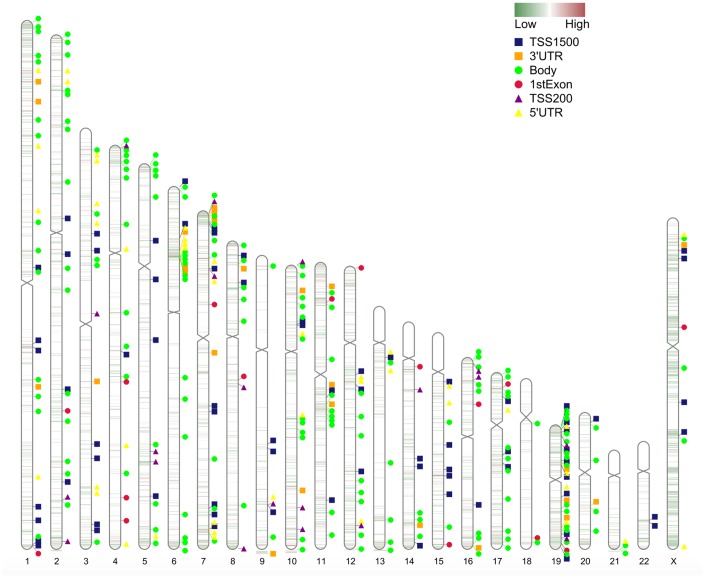
**Chromosome distribution of differentially methylated intergenic CpGs.** The plot presents the distribution of differential intergenic CpG sites at 22 autosomes and the X chromosome. Regions in red are hypermethylated regions, and regions in green are hypomethylated regions. The value is the logFC of the M value among asymptomatic and symptomatic patients.

The chromosome distribution of differentially methylated intergenic CpGs is shown in Figure 3. Regions in red are hypermethylated regions, and regions in green are hypomethylated regions.

### Functional enrichment analysis for DMGs

**Figure 4:**
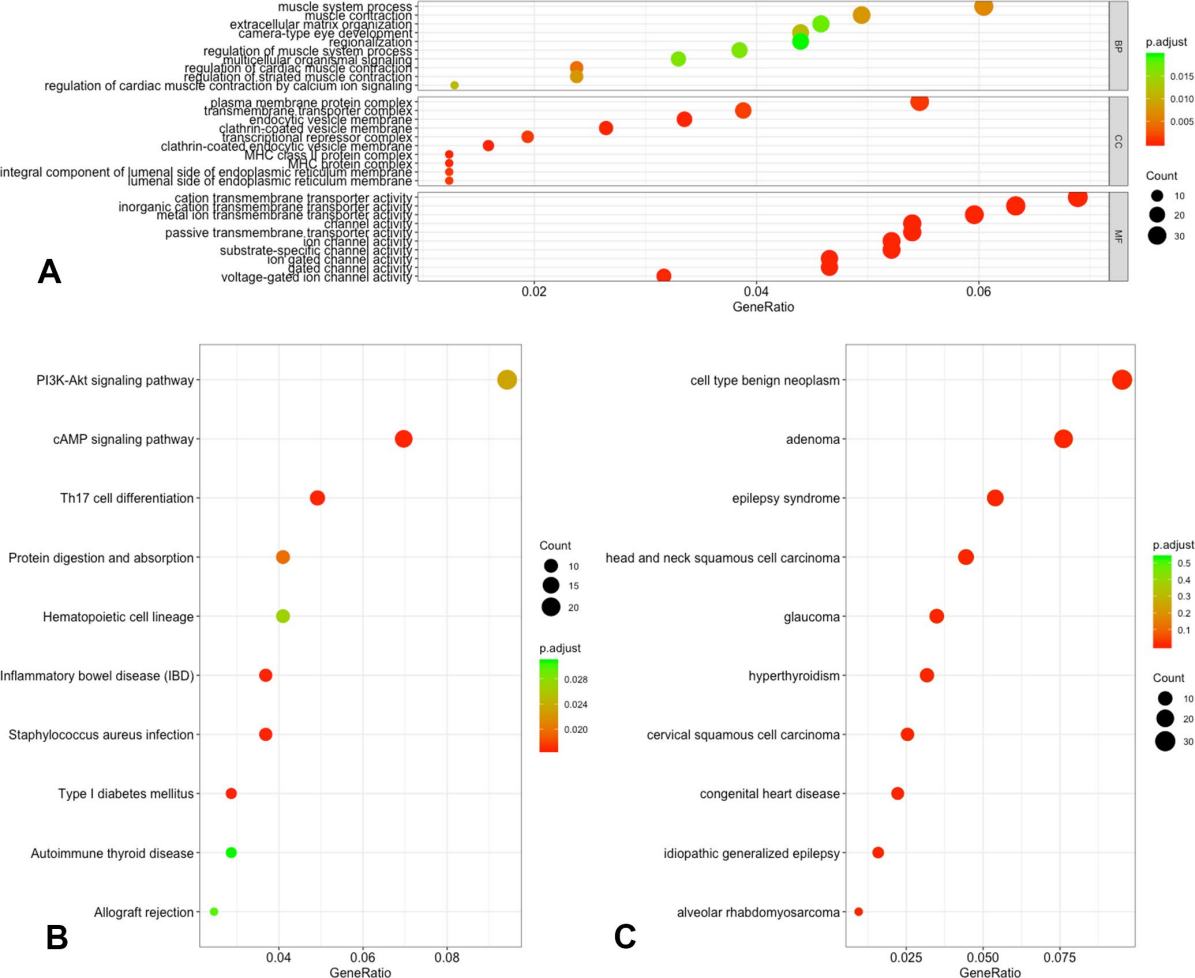
**Functional annotation of DMGs.** (**A**) GO analysis of DMGs; (**B**) KEGG analysis of DMGs; (**C**) DO analysis of DMGs.

As shown in Figure 4, the most important items in the development of IS and all of the detailed information can be found in Supplementary Table 2. In the analysis of GO functions, 50 biological processes, 58 cellular components as well as 46 molecular functions were recognized with adjusted-*P* < 0.05. Approximately 18 pathways were enriched in the KEGG pathway analysis, and none of the DO items were analyzed for the screened DMGs with adjusted-*P* < 0.05. However, if the threshold value was set at *P* < 0.05, we could include 39 DO items for further analysis.

Among these terms, GO:0035637 multicellular organismal signaling, GO:0007265 Ras protein signal transduction, hsa04659 Th17 cell differentiation, hsa05321 Inflammatory bowel disease (IBD), hsa04024 cAMP signaling cascade, hsa04151 PI3K-Akt signaling cascade and hsa05320 Autoimmune thyroid disease were confirmed in previous references to be associated with IS, and the genes associated with these terms were chosen for additional evaluation.

### PPI network construction and submodule analysis

**Figure 5:**
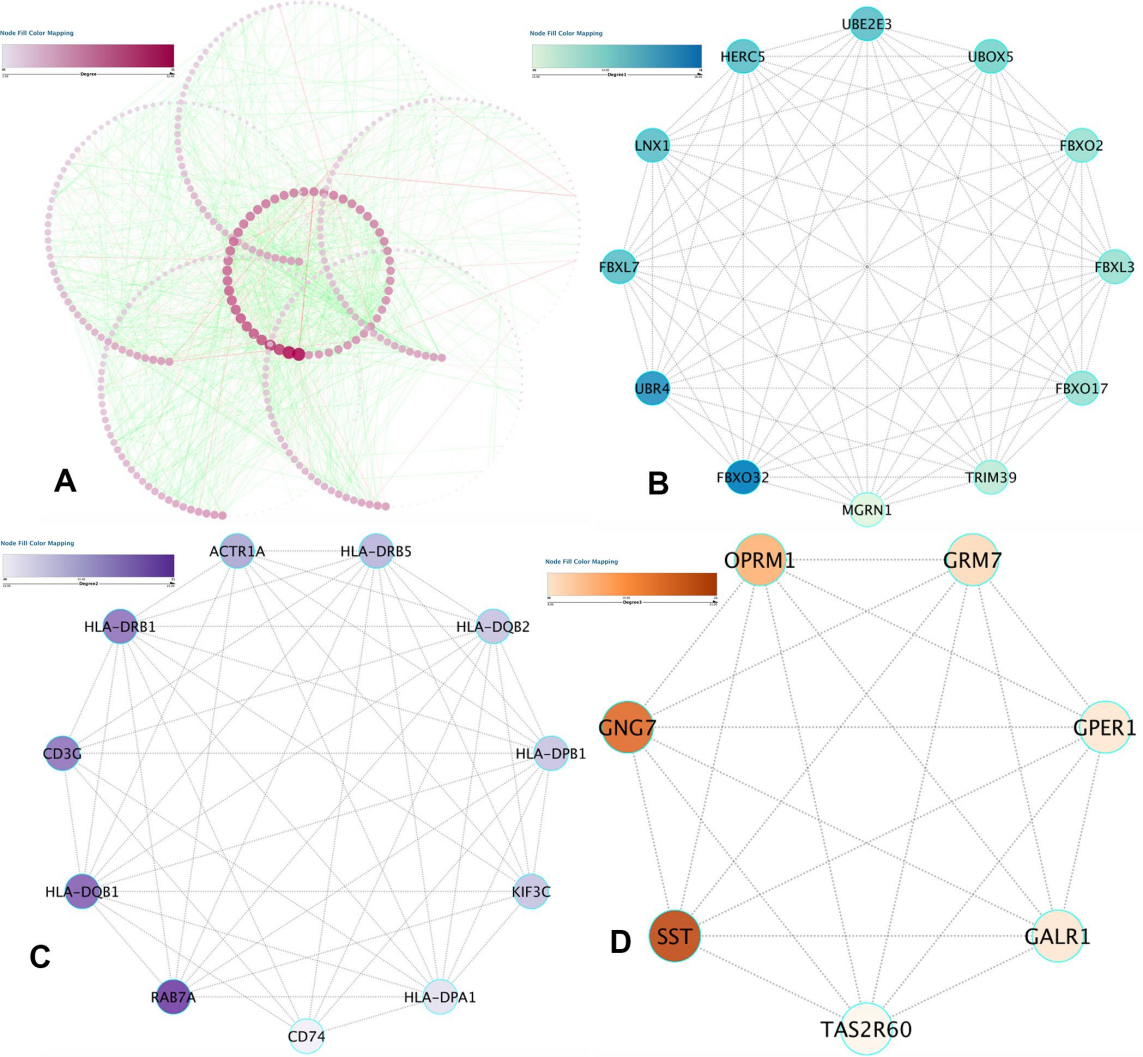
**PPI network construction and hub item identification.** (**A**) PPI network of the selected DMGs. Edges stand for the interaction between two genes. The significant modules recognized in the PPI network by the molecular complex detection technique with a score of > 7.0. (**B**) Molecular-1 with MCODE = 12; (**C**) Molecular-2 with MCODE = 9.8; (**D**) Molecular-3 with MCODE = 7.6. A degree was utilized to explain the prominence of protein nodes in the network; dark colors show a high degree, and light colors present a low degree.

Data analysis was done on the STRING database, which revealed 1463 protein pairs and 552 nodes with a combined score > 0.9. Figure 5A shows the network analysis in Cytoscape software. When detected by the MCODE app, three modules with a score > 7 were identified and are represented in Figure 5B–5D. After synthesizing the data of GO, DO, and KEGG analyses, we selected 2 DMGs (HLA-DRB1 and HLA-DQB1) as hub genes correlated to the onset of IS with a high degree and included them in the submodule analysis at the same time.

### Hub gene validation

**Figure 6:**
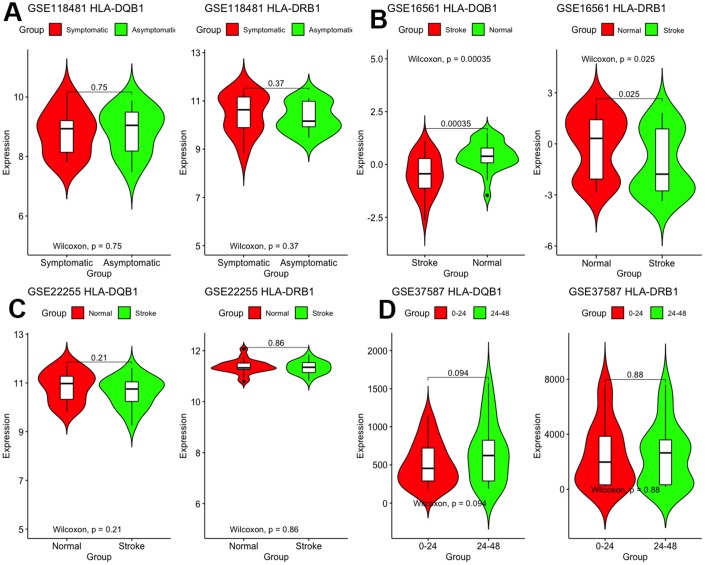
**Validation of mRNA expression of interest in different datasets.**

First, we validated these two genes in different microarray datasets. As shown in Figure 6, differences between the two genes were found only in GSE16561 but not in other datasets. The expression of *HLA-DRB1* and *HLA-DQB1* was lesser in stroke patients than in normal controls (*P* < 0.05).

Then, we implemented a correlation analysis to distinguish if DNA methylation caused IS through regulation of gene expression. Generally, the increase in DNA methylation affects the binding of transcription factors, leading to abnormal gene transcription, usually inhibiting transcription and resulting in the downregulation of gene expression. However, it was not absolute. In any case, changes in methylation can cause changes in gene expression.

**Figure 7:**
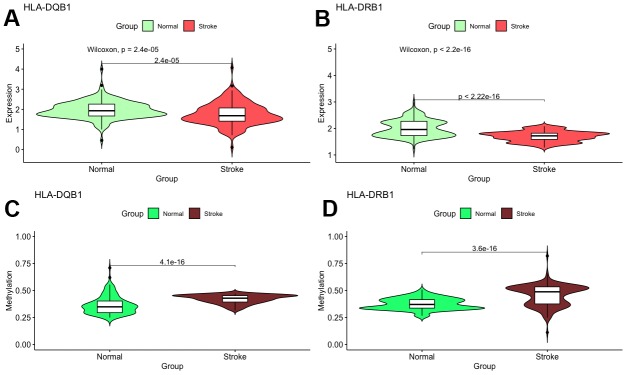
**Validation of mRNA expression as well as DNA methylation of interest between IS and healthy samples.**

**Figure 8:**
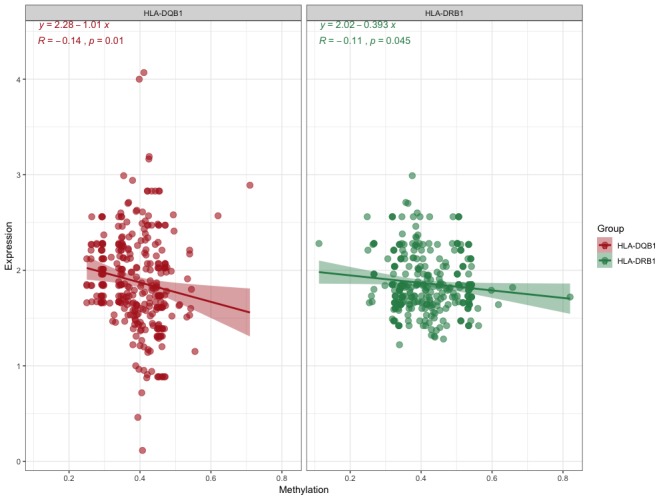
**Correlation analyses for DNA methylation and mRNA expression.**

With these conditions, we selected these 2 significantly correlated methylation–mRNA pairs for testing a total of 322 samples (161 healthy control and 161 IS). The 322 validation samples were coordinated for age and sex. The weight, BMI, waist circumference, smoking status, serum TC and LDL-C levels were higher in IS patients compare to controls (Table 2). Initially, we examined the methylation of these 2 genes in two samples and established that all of them showed increased hypermethylation compared with that in the IS group (Figure 7C–7D). Next, we found that the relative expression of these two genes was lower in stroke samples (Figure 7A–7B). This result also coincided with GSE16561. Then, we implemented correlation analysis among DNA methylation and gene expression in the similar samples and established that *HLA-DRB1* and *HLA-DQB1* gene methylation levels were negatively associated with their expression (Figure 8). This result indirectly confirmed that modifications in the methylation of the promoter region of *HLA-DRB1* and *HLA-DQB1* caused atypical gene expression, causing the beginning of IS.

**Table 2:** Comparison of demographics, lifestyle characteristics and serum lipid levels between the normal and IS groups.

**Parameter**	**Control**	**IS**	***test-statistic***	***P***
Number	161	161		
Male/female	49/112	51/110	0.058	0.810
Age (years)^1^	58.21±9.45	58.88±9.23	0.824	0.406
Height (cm)	156.13±6.92	155.58±7.12	1.594	0.222
Weight (kg)	51.94±7.22	60.73±11.44	18.439	1.23E-005
Body mass index (kg/m^2^)	28.21±3.08	31.43±6.17	28.204	2.52E-008
Waist circumference (cm)	71.41±6.53	88.01±9.96	22.122	6.17E-005
Smoking status [*n* (%)]	42(26.3)	57(35.8)	3.282	0.070
Alcohol consumption [*n* (%)]	39(24.3)	41(25.8)	0.067	0.796
Systolic blood pressure (mmHg)	127.43±15.13	129.47±22.18	4.533	0.023
Diastolic blood pressure (mmHg)	80.51±10.21	83.24±14.13	5.223	0.015
Pulse pressure (mmHg)	49.67±12.13	50.27±13.24	1.452	0.263
Glucose (mmol/L)	5.84±1.53	5.92±2.73	2.783	0.137
Total cholesterol (mmol/L)	4.94±1.13	5.38±1.26	7.333	0.010
Triglyceride (mmol/L)^2^	1.49(0.51)	1.53(1.22)	2.137	0.187
HDL-C (mmol/L)	1.56±0.43	1.21±0.38	7.137	0.011
LDL-C (mmol/L)	2.96±0.81	3.73±1.92	11.228	3.53E-004
ApoA1 (g/L)	1.22±0.21	1.13±0.24	0.382	0.509
ApoB (g/L)	0.84±0.19	0.93±0.30	1.568	0.223
ApoA1/ApoB	1.68±0.51	1.65±0.53	0.088	0.722

*IS,* ischemic stroke; *HDL-C*, high-density lipoprotein cholesterol; *LDL-C*, low-density lipoprotein cholesterol; *Apo*, Apolipoprotein. ^1^Mean ± SD determined by *t*-test. ^2^Because of nonnormally distribution, the triglyceride value was presented as median (interquartile range), and the difference between the two groups was determined by the Wilcoxon-Mann-Whitney test.

## DISCUSSION

Ischemic stroke (IS) is a complicated disorder with great mortality as well as long-term disability outcomes. In spite of many concerns about stroke risk factors as well as prophylactic treatment, the number of stroke cases has been increasing recently, probably due to the increasing age of the population [12]. The pathogenesis of stroke involves many different disease processes and interactions between the central nervous system and environmental, systemic, genetic and vascular factors. Approximately 80% of strokes were ischemic, and the other 20% were hemorrhagic. At present, our studies focused on IS and its most usual subtypes: cardiac aortic embolism (CE), arteriolar disease (SAD) and arteriosclerosis (LAA) [13]. A large body of evidence from twin, family, and animal model studies [14] suggested that genetic risk components are connected with stroke; additionally, latest genome-wide association reports have found novel variants related to IS along with IS subtype-specific genetic variations [15]. These genetic factors can lead to traditional risk factors, for example homocysteine concentrations (with recognized genetic components) or diabetes, and hypertension might interrelate with environmental factors, such as smoking and drinking, or lead to intermediary phenotypes, such as atherosclerosis. Epidemiological data have offered several lines of proof for a genetic component of the disease, but with limited awareness of its incidence and characteristics. Hence, it was necessary to uncover novel biomarkers for stroke risk. Moreover, the participation of epigenetics was still mostly unidentified.

Epigenetics is receiving increasing attention as it might contribute to the research of complex diseases and might also produce valuable biomarkers. Epigenetic mechanisms, for example DNA methylation, control higher-order DNA structure as well as gene expression. In recent years, with the continuous development of technology, the correlation between genome-wide methylation and IS has gradually been confirmed [16]. Shen et al. found that methylation of *MTRNR2L8* was a diagnostic biomarker for stroke and may also be a potential therapeutic target [17]. Fujii et al. found that eating a large amount of vegetables every day can reduce the methylation of the *ABCA1* gene and promote the reverse flow of cholesterol, weakening the trend of atherosclerosis. Interestingly, the study was validated only in women [18].

HLA, which is defined as the human major histocompatibility complex, functions as an essential element of the immune system. The major HLA antigens are HLA-A, HLA-B, HLA-C, HLA-DR, HLA-DP, and HLA-DQ. HLA molecules have an imperative role in the transplantation reaction and immune response to various immunogens as well as infections [19]. In addition, there was evidence that HLA was associated with ischemic disease, atherosclerosis and cancer [20]. Murali et al had found that HLA-DRB1*/DQB1* alleles and haplotypes strongly predispose South Indian population to ischemic stroke. But large sample size or the meta-analysis are needed to explain the exact mechanism of associations of HLA gene(s) with IS [21]. Moreover, the HLA complex gene was a genetic risk factor for idiopathic ischemic stroke in children, suggesting that HLA molecules were involved in ischemic stroke [22]. HLA-DRB1 and HLA-DQB1 belong to the HLA class II beta chain paralogs. These class II molecules form a heterodimer comprising of an alpha and a beta chain, both attached in the membrane. It has a vital function in the immune system by presenting peptides resulting from extracellular proteins. Recently, more and more studies have supported that atherosclerosis as a chronic inflammatory disease, and its inflammatory response was related to immune system dysfunction. When inflammation occurred, vascular endothelium was damaged, and a large number of macrophages engulfed lipids and contributed to the formation of arterial plaques. *HLA-DRB1* and *HLA-DQB1* genes have been proved to play an important role in the process of immune inflammation. Changed in their expression levels may eventually resulted in inflammation of intracranial arteries, leading to IS. Previous studies have confirmed that these two genes are clearly associated with IS [23, 24].

We used other datasets for verification and found that these two genes were significantly expressed at low levels in the IS population. Interestingly, we validated this conclusion in only one dataset, and there was no difference in the relative expression of these two genes within 24 hours and 24–48 hours after symptom onset. The reason for this was related to the small sample size of the dataset. Therefore, we found IS patients and healthy people in order to extract peripheral blood and at the same time, to verify the methylation and relative expression of the promoter regions of these two genes. We found that when the methylation of the promoter region increased, the gene expression decreased significantly, and methylation and expression were clearly correlated. The above conclusions are consistent with previous research results.

We have to admit the limitations of this study. First, the validation sample is small, and patients in this study are from two hospitals; hence, there may be differences with patients from diverse areas and of different races. Second, the precise mechanism of the (DNA methylation)–mediator (mRNA)–outcome (IS) network for controlling the pathological processes of IS has not been abundantly confirmed in *vivo* or in *vitro*.

In brief, we acquired the GSE66500 dataset from GEO and identified DMPs and genes. We chose 2 DMGs for validation in additional datasets and acquired 322 samples (161 IS patients and 161 healthy controls). HLA-DRB1 and HLA-DQB1 were found to exhibit hypermethylation and downregulated gene expression in IS patients. In addition, correlation analysis revealed that DNA methylation instigated IS through the regulation of gene expression.

## MATERIALS AND METHODS

### Gene expression omnibus database

GSE66500 [25] was retrieved from the GPL13534 Illumina Infinium HumanMethylation 450 BeadChip for Genome-wide DNA methylation analysis. This dataset consisted of 19 asymptomatic and 19 symptomatic patients, and the sample source was carotid plaque. All data processing and differential methylation positions (DMPs) were identified in GEO2R. Moreover, CpG sites on the sex chromosomes were eliminated to prevent sex-specific methylation bias. DMPs positioned in the gene region were allocated to the analogous genes that were defined as differentially methylated genes (DMGs). The threshold was set at |log_2_ fold-change| (Δβ) > 0.05 and *P* < 0.05. GSE118481, GSE16561 [26], GSE22255 [27] and GSE37587 [28] were also acquired from the GEO database and utilized as the validation sample. From these datasets, the functions of hub genes were verified from different dimensions. We employed the Affy package in R [29] to transform CEL files into an expression value matrix and RMA methods to normalize the matrix. Subsequently, we converted the probe data to gene with the Bioconductor package in R software [30]. If a gene corresponded to several probes, we chose the mean expression value for further analysis.

### Functional enrichment analysis

We compared obese subjects with controls to explore the differentially expressed genes (DEGs) with the limma package in R [10]. The threshold values were set at |log_2_ fold-change| ≥ 2 and *P* < 0.05. Then, we used GEO2R to identify the differentially methylated positions (DMPs) by comparing the normal and obese subjects. DMPs positioned in the gene region were allocated to the analogous genes that were defined as differentially methylated genes (DMGs). The threshold was set at |log_2_ fold-change| (Δβ) > 0.05 and *P* < 0.05. Subsequently, we matched DEGs to DMGs, and simply the matched genes (DEMGs) were chosen for additional examination.

### Functional enrichment analysis

Analyses on large-scale transcription data or genomic data were generally done depending on functional enrichment analyses. These include disease ontology (DO), Kyoto Encyclopedia of Genes and Genomes (KEGG) pathway as well as gene ontology (GO) analyses. In the current study, we used clusterProfiler [31] along with the DOSE [32] package in R to analyze DMGs. The threshold for the analysis was set at adjust-*P* < 0.05 along with false discovery rate (FDR) < 0.05. To further determine the location of hub sites in the dataset, shrunken centroid classifier analysis was performed with the “pamr” package in R [33].

### Protein-protein interaction (PPI) network creation as well as module analysis

The protein prediction as well as experimental interactions was examined by the STRING database (version 11) [34]. Gene fusion, co-expression experimentations, databases, text mining, neighborhoods as well as co-occurrence are the usual prediction approaches for the database. Additionally, a combined fraction was utilized to demonstrate the interaction of proteins. In the current study, DMGs were mapped to PPIs, and a combined score > 0.9 was considered as the cutoff value [35] to evaluate main genes in the network. Degrees were used as a vital way to present the role of protein nodes. Network modules are one of the mainstays of protein networks and might have precise biological impacts. The Molecular Complex Detection (MCODE) of the Cytoscape software (version 3.71) [36] was utilized to recognize the main clustering modules as well as the most prominent clustering modules. After that, we selected EASE ≤ 0.05 and count ≥ 2 for the cutoff value and an MCODE score > 7 as the threshold for the additional succeeding evaluation.

### Validation of DMGs of interest

First, we validated the hub genes in other expression datasets to explore the relationship between hub genes and IS in different dimensions. GSE118481 reflected the expression level of core genes in carotid plaques between cerebral ischemic symptoms and asymptomatic conditions. GSE16561 and GSE22255 indicated whether the comparative expression of hub genes was different among stroke patients and normal controls. GSE37587 further evaluated the relative expression level of hub genes in patients with cerebral infarction at different time points after symptom onset. Next, we sought out stroke and healthy control groups to extract peripheral blood for core gene validation.

### Sample authentication and diagnostic standards

A total of 322 subjects with complaints related to the brain at the First People’s Hospital of Nanning City from Jan. 1, 2015, to Dec. 31, 2017, were recruited. The blood biochemistry levels were 3.10–5.17 (TC), 0.56–1.70 (TG), 0.91–1.81 (HDL-C), 2.70–3.20 (LDL-C) mmol/L, 1.00–1.78 (ApoA1), 0.63–1.14 g/L (ApoB), and 1.00–2.50 (ApoA1/B), which were stated as the standard values [37]. All of the selected IS patients received a thorough neurological checkup along with brain magnetic resonance imaging. IS was diagnosed as per the International Classification of Diseases (9^th^ Revision). Subjects with an embolic brain infarction, stroke triggered due to inflammatory disease, cardioembolic stroke, autoimmune disease, or serious chronic conditions were omitted from the current study [38]. The controls were evaluated to be free of IS by questionnaires, medical history, along with medical investigation. All subjects were from the Han population in Guangxi, China. A standard questionnaire was utilized to determine overall information along with medical history from all patients. This study was approved by the Ethics Committee of the First People’s Hospital of Nanning City and Liuzhou People’s Hospital (No. Lunshen 2009-Guike018; Jan. 7, 2009). Informed consents were acquired from all participants [39].

### Quantitative DNA methylation

Genomic DNAs from the 322 peripheral blood samples were obtained with a TaKaRa MiniBEST Universal Genomic DNA Extraction Kit Ver.5.0. DNA concentrations were determined by means of a NanoDrop2000 spectrophotometer (USA). The methylation levels of CpG sites were assessed through pyrosequencing. PyroMark Assay Design software (Qiagen) was utilized to design precise sets of primers for CpG PCR amplification as well as sequencing. The primers can be found in Supplementary Table 3. All protocols for bisulfite conversion, PCR and pyrosequencing were previously described [10]. DNA methylation of the hub gene promoter was computed by MassARRAY EpiTYPER assays (Sequenom, USA). Sequenom EpiDesigner software was used to design the primers. Procedures for methylation evaluations as well as quality controls have been published previously [40].

### Real-time PCR analysis

Total RNA from the 322 peripheral blood samples was extracted by means of an Axygen RNA Isolation Kit (USA) as per the supplier’s protocol. RNA concentrations were determined by means of a NanoDrop2000 spectrophotometer (USA). cDNA was synthesized from 1 μg of total RNA using a PrimeScript 1^st^ strand cDNA Synthesis Kit (TaKaRa, China) as per the supplier’s instructions. Real-time polymerase chain reactions were carried out to evaluate the mRNA expression levels of hub genes using SYBR Premix Ex Taq II (TaKaRa, China) by using a 7500 Real-Time PCR system (Applied Biosystems, USA). GAPDH served as an internal control. The primer sequences are mentioned in Supplementary Table 4.

### Statistical analysis

Data analyses were done using SPSS 22.0 (SPSS Inc. USA) and Prism 8.0 (GraphPad Software). Chi-square analysis was implemented to evaluate differences in ratios amongst groups. Continuous data are presented as the means ± SD for those that were normally distributed; the median and interquartile ranges of TG were not normally distributed. The Mann-Whitney nonparametric test and Kruskal-Wallis test were utilized to compare continuous data sets. R software (version 3.6.0) was utilized for further bioinformatics analysis. To define if the methylation level was related to gene expression, we conducted a correlation test between methylation and expression using the ggplot2 package in R.

## Supplementary Material

Supplementary Table 1

Supplementary Table 2

Supplementary Tables 3 and 4
