# Supplementary material for: Association between promoter DNA methylation and gene expression in the pathogenesis of ischemic stroke
Source: Aging (Albany NY). 2019 Sep 17;11(18):7663–77. doi: 10.18632/aging.102278 (PMC6781986; doi:10.18632/aging.102278)
Supplement: Supplementary Tables 3 and 4 [file aging-11-102278-s003.pdf]

SUPPLEMENTARY TABLES

Please browse Full Text version to see the data of Supplementary Tables 1 and 2.

Supplementary Table 1. The details of DMPs.

Supplementary Table 2. Functional annotation for DMGs.

Supplementary Table 3. The primers designed for target bisulfite sequencing.

	F	R
HLA-DRB1	atggcctgggcacaatgttaGAGTTCCCAAGACTTGCCCA	ctccagcatggtgtgtctga GGGACACCCGACGTAAGTG
HLA-DQB1	tctttgcgggcttctggaat GATTTCAGAGACCTCGCCCC	acgccctgatccctctaagt GATTCCAGAAGCCCGCAAAG

Supplementary Table 4. The primers designed for RT-PCR.

	F	R
HLA-DRB1	AATGTGCACTTACGTCGGGT	CCCTGAGTGAGACTTGCCTG
HLA-DQB1	TTTGCGGGCTTCTGGAATCT	GGGATTTCATGCGAGAACGC
GAPDH	GCACCGTCAAGGCTGAGAAC	TGGTGAAGACGCCAGTGGA
